# The diagnostic performance of point-of-care tests for serum amyloid A and C-reactive protein in lame sows

**DOI:** 10.1186/s13028-026-00857-6

**Published:** 2026-03-25

**Authors:** Nadia Jakobsen, Inge Larsen, Nicolai Rosager Weber, Ken Steen Pedersen

**Affiliations:** 1 Department of Veterinary and Animal Sciences, Faculty of Health and Medical Sciences, University of Copenhagen, Frederiksberg C, Denmark; 2 Danish Agriculture & Food Council F.m.b.A, Copenhagen, Denmark; 3 Ø-Vet A/S, Naestved, Denmark

**Keywords:** C-reactive protein, Diagnostic performance, Evaluation, Point-of-care tests, Sandwich ELISA, Serum amyloid a

## Abstract

**Background:**

For acute phase proteins to be implemented in routine diagnostics for pig herds, a fast, reliable, and portable point-of-care test is required. Therefore, this study aimed to evaluate the diagnostic performance of two point-of-care tests on lame gestating sows. The two tests were the Cobas b101 CRP test for C-reactive protein and OmniChek^TM^-SAA for serum amyloid A. The reference test was an enzyme-linked immunosorbent assay (ELISA).

**Results:**

The C-reactive protein point-of-care test had a concordance with the ELISA results of 0.12 (95% confidence limits: 0.12–0.23) and a mean difference of − 30.98 µg/mL (Limits of agreement: 27.45 and − 89.40 µg/mL), with a clear proportional bias. The serum amyloid A point-of-care test was only in agreement with the ELISA results in two out of 49 samples. The serum amyloid A test overestimated the serum amyloid A levels, whereas the C-reactive protein point-of-care test underestimated the C-reactive protein levels.

**Conclusions:**

Currently, the two point-of-care tests cannot be reliably used to determine acute phase protein levels in blood samples from lame gestating sows.

**Supplementary Information:**

The online version contains supplementary material available at 10.1186/s13028-026-00857-6.

## Background

A point-of-care test (POCT) is a diagnostic test that can be performed at or near the point of care [1]. To be relevant for in-herd diagnostics, the POCT should be portable, have a short run-around time, be minimally invasive, and require no to limited laboratory equipment. Point-of-care testing is common in human medicine, and the tests are frequently used in primary care to detect specific pathogens, e.g., group A streptococci [2]. Further, POCTs are used to guide antibiotic treatment through biomarkers such as procalcitonin and C-reactive protein (CRP) [2–5]. In pigs, multifactorial conditions like lameness, diarrhoea, and respiratory infections, where clinical signs are not indicative of aetiology, are frequently encountered. For these conditions, rapid pathogen-specific tests are not available, or the available methods are time-consuming [6]. Hence, antibiotic treatments are initiated without identifying the causative agents, which in the case of lameness in sows and finishers have led to treatment of non-infectious cases [7]. Using acute phase protein levels to guide antibiotic treatment could perhaps, as seen in humans [4, 8] and dogs [9, 10], ensure that treatment with antibiotics is only initiated when indicated. Several studies have shown that common diseases and conditions in pigs are associated with an increase in acute phase protein levels [11–13]. However, acute phase proteins have not yet been implemented in the diagnostic toolbox for pig veterinary practitioners. One explanation could be the lack of standardized, commercially available, and reliable POCTs for porcine acute phase proteins. Hence, this study aimed to validate commercially available acute phase protein-based POCTs. In pigs, the moderate to major acute phase proteins are C-reactive protein, haptoglobin, pig-major acute phase protein (Pig-MAP), and serum amyloid A (SAA) [14]. No commercially available POCTs were found for Pig-MAP or haptoglobin. Therefore, the objective of the study is to evaluate the diagnostic performance of two commercially available POCTs on lame gestating sows without signs of other diseases. One POCT targeted CRP, and the other targeted SAA. The tests were performed in-herd on lame gestating sows.

## Methods

### Study design

The validation study was part of a larger study focusing on acute phase protein levels in lame and healthy sows from 12 conventional Danish sow herds on Zealand. The point-of-care tests were tested on blood from lame sows, rendering 49 samples for the validation study. The herds had loose housing in the gestation unit, with a mixture of stable and dynamic groups. The pens contained 20–400 sows and had floor or electronic sow feeding. The full protocol can be found in [15]. In each herd, the assessor entered gestation pens containing approximately 200 sows and screened half of the sows for signs of disease and lameness. A sow was available for inclusion in the study if it was free from all signs of disease except lameness. After the initial screening, information regarding parity, expected farrowing date, and treatment in the gestation unit was extracted, and up to five lame sows were chosen randomly. Hereafter, a thorough clinical evaluation was performed focusing on behaviour (activity level, orientation skills and interest in surroundings), posture and movement (head position, claw injuries, ability to stand, equal presentation of hind and front legs, degree of lameness and affected limbs), well-being (body condition, rectal temperature, skin colour including vulva), signs of respiratory, reproductive or gastro-intestinal disease (coughing, sneezing, discharge, diarrhoea, prolapse, vulva bites or chronic mastitis) and signs of inflammation on the body and affected limbs (inflamed wounds, redness, ulcers and swellings). Blood was sampled into two tubes from the vena jugularis. The sample was taken using an 18-G needle (BD, Mississauga, Canada), while the sow was restrained using a snout snare. The blood was collected into one K_3_EDTA vacutainer (BD, Mississauga, Canada) for haematology analysis and POCT validation and one serum vacutainer (BD, Mississauga, Canada) for the acute phase protein ELISAs. At the time of sampling, the sows had not received treatment for the lameness in the gestation unit and were mildly to severely lame on one to several legs. Sixteen out of 49 sows had signs of inflammation, e.g., redness, wounds, or swelling on the affected limb(s). The average parity was 2.2, and the sows were on average 9.5 weeks into the gestation.

### Point-of-care tests

The two POCTs used in this study were OmniChek^TM^-SAA (Accuplex Diagnostics, Kildare, Ireland) and Cobas b101 CRP (Roche Diagnostics, Copenhagen, Denmark). The tests were performed according to the manufacturer’s instructions, with the exception that blood from the K_3_EDTA vial was used as opposed to collecting blood directly from the ear vein/capillaries of the sow. This is a method that was described as compatible with both POCTs. In short, the OmniChek^TM^-SAA was performed by extruding 3 µL of blood from the K_3_EDTA vial and adding it to the sample applicator of the test. Hereafter, three drops of buffer were added from the dropper bottle, and the results were read after 10 min. For the Cobas b101 CRP, the instrument was turned on, and an optical check disc was inserted to ensure that the optical functions were normal before each test round. Before inserting the test cassette, the Sow ID, date, and type of test were registered on the instrument. Hereafter, 12 µL of whole blood was added to the test cassette from the K_3_EDTA vial using a serum pipette (Sarstedt AB, Helsingborg, Sweden). The cassette was inserted into the Cobas b101 CRP instrument. After approximately 3 min, the test result was read. In case of invalid results, the procedure was redone. The POCTs were not run in duplicate.

### ELISA test (reference test)

A commercially available sandwich ELISA (Phase SAA assay, Tridelta Development Ltd., Kildare, Ireland) with a detection limit of 1.56 µg/mL (porcine SAA equivalents) was used for the determination of SAA serum concentrations. The assay has been described elsewhere [16]. The assay was performed according to the manufacturer’s instructions, except that the lowest dilution was 1:100 as opposed to 1:500 as recommended. C-reactive protein serum concentrations were determined by a sandwich ELISA as previously described [17]. The assay had a detection limit of 1.42 µg/mL (human equivalents) and used dendrimer-coupled cytidine diphosphocholine, polyclonal rabbit anti-human antibodies with cross-reactivity towards porcine CRP [18] and peroxidase-conjugated goat anti-rabbit antibody (DAKO Aps, Glostrup, Denmark). Plates were developed with a tetramethylbenzidine (TMB) peroxide colour substrate (Kem-En-Tec Nordic Aps, Taastrup, Denmark) and read using an automatic plate reader (Thermo Multiskan Ex spectrophotometer, Thermo Scientific, Waltham, MA, USA). All samples were run in duplicate.

### Sample size

The Cobas b101 CRP (Roche Diagnostics, Copenhagen, Denmark) is a quantitative test, and the sample size was calculated using an expected correlation of 0.5, a desired power of 0.80, and an alpha level of 0.05. The calculated minimum sample size was 29. The second point-of-care test was the OmniChek^TM^-SAA (Accuplex Diagnostics, Kildare, Ireland), which is semi-quantitative. The performance was evaluated based on the sensitivity and specificity, and the sample size was calculated with an expected sensitivity of 0.60, specificity of 0.70, power of 0.80, and alpha-level of 0.05, rendering a sample size of 42. Based on the sample size estimates, it was decided to include all blood samples from the lame sows (*n* = 49).

### Statistical analysis

The acute phase protein levels in each blood sample were determined in duplicate, and an average was calculated and compared to the results offered by the POCT. For the CRP analysis, the Cobas b101 CRP provided a numerical value between 3 and 400 mg/L. If samples were below 3 mg/L, the result was treated as a 0 during the statistical analysis. No values above 400 mg/L were found by the POCT. The units were changed from mg/L to µg/mL for the statistical analysis, which was conducted in R version 4.2.2. The correlation between the standard method (Sandwich ELISA) and the point-of-care test (Cobas b101 CRP) was performed by Lin’s concordance correlation coefficient (CCC) [19, 20]. Agreement was evaluated using a Bland-Altman plot [21]. The OmniChek^TM^-SAA is a semiquantitative lateral flow assay, which categorises the SAA levels into four categories; 3 lines corresponded to a SAA level of < 10 µg/mL, 2.5 lines corresponded to 10–50 µg/mL SAA, 1.5 to 2 lines corresponded to 50–400 µg/mL SAA and 1 line corresponded to a SAA level of > 400 µg/mL. To compare the lateral flow assay results to the results provided by the Sandwich ELISA, the ELISA results were transformed into the levels provided by the OmniChek^TM^-SAA test. For simplicity, the result 1.5 to 2 lines was denoted 2 and contained SAA values from 50 to 400 µg/mL. The analysis was conducted in R version 4.2.2.

## Results

### Agreement between ELISA and Cobas b101 CRP for estimating C-reactive protein levels

**Fig. 1:**
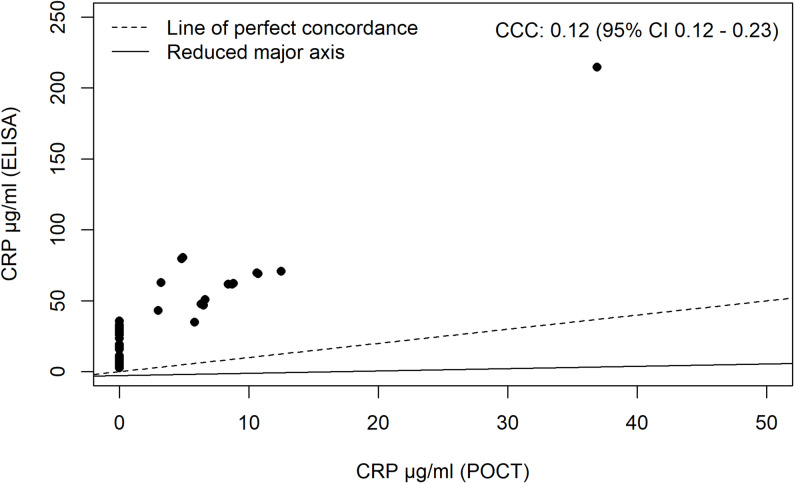
Lin’s concordance correlation coefficient plot for the C-reactive protein (CRP) levels. The y-axis depicts the CRP levels from the ELISA analysis, and the x-axis contains the CRP levels from the POCT. The dotted line represents the line of perfect concordance, and the solid line the reduced major axis. The concordance correlation coefficient (CCC) and the confidence interval (CI) are depicted at the top right corner

Lin’s concordance correlation coefficient was calculated, and the plot of the concordance can be found in Fig. 1. As can be seen from the plot, the concordance correlation coefficient was 0.12 with the confidence interval ranging from 0.12 to 0.23. Furthermore, the bias correction factor was 0.19, indicating a poor positive correlation between the two methods. This is also apparent by the “line of perfect concordance” and the “reduced major axis” having different slopes and placement in the plot; for perfect concordance, the lines should be indistinguishable.

**Fig. 2:**
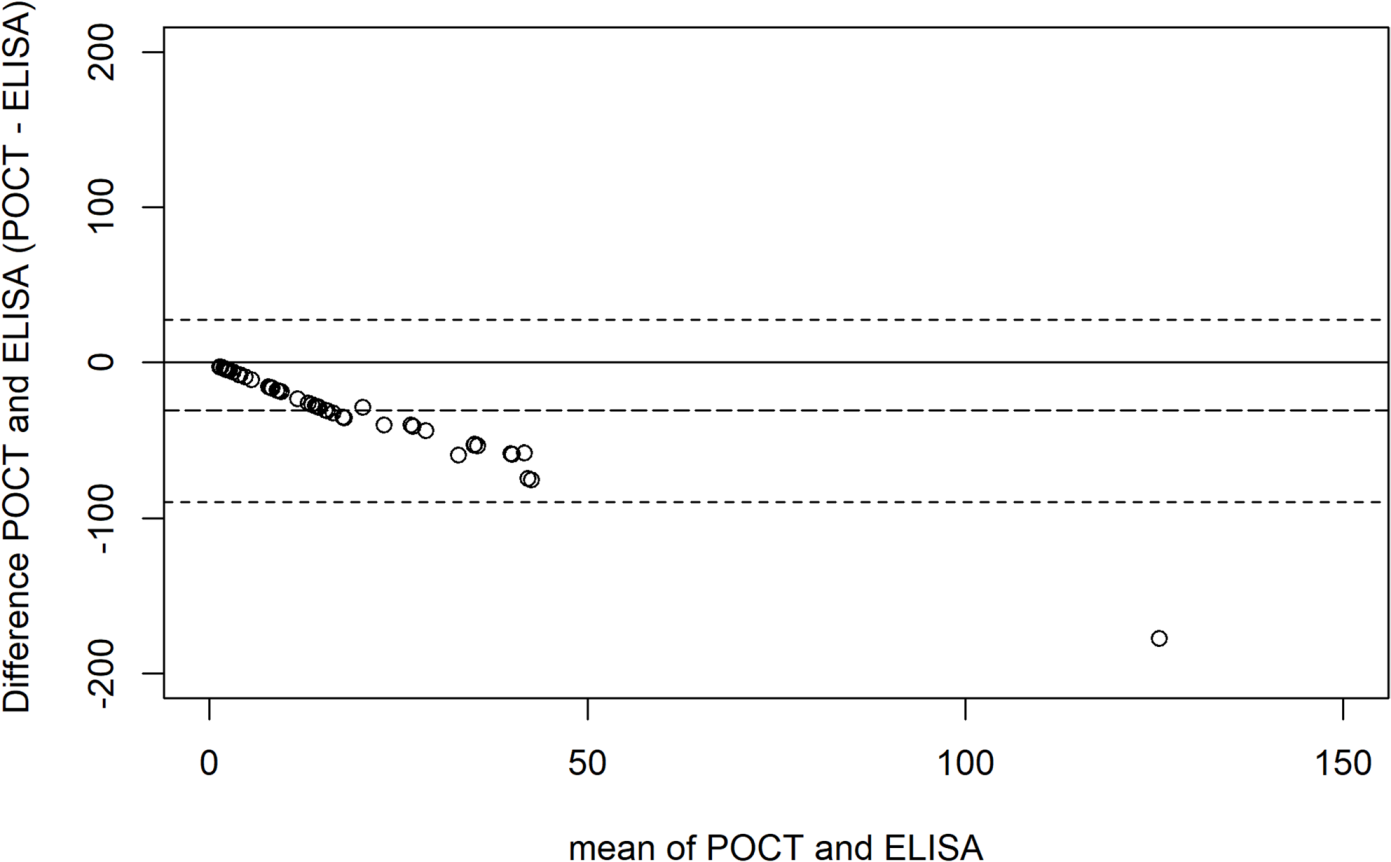
Bland-Altman plot of the difference in C-reactive protein (CRP) (ELISA- Cobas b101 CRP). The plot visualises the limits of agreement (The two dashed lines) calculated as d ± 2s. d = mean difference and s = SD of the difference. The Y-axis depicts the mean difference in CRP levels (POCT – ELISA), and the X-axis the mean CRP level from the POCT and the ELISA test. The long-dashed line corresponds to the mean difference

The agreement between the point-of-care test and the reference test was evaluated using a Bland-Altman plot with the mean difference plotted against the mean of the two tests. The Bland-Altman plot can be seen in Fig. 2. The mean difference was – 30.98 µg/ml, and the limits of agreement were 27.45 and − 89.40 µg/mL. The data suggested that the CRP levels provided by the POCT were lower than those found by the sandwich ELISA and that the POCT missed a lot of the samples in the 0 to 35 µg/mL range. Additionally, the underestimation increased with higher CRP levels. The data can be found in Additional file 1.

### Agreement between ELISA and OmniChek^TM^-SAA for estimating serum amyloid A level

The LOD was 1.56 µg/mL for the SAA ELISA, which meant that 36 out of 49 blood samples could not be quantified by the ELISA because they were too low. Of the 16 samples that were quantified, 12 were below 10 µg/mL, three were between 10 and 50 µg/mL, and one was above 400 µg/mL. The results of the POCT and the ELISA can be found in Additional file 1.

The ELISA results were categorized as previously described, and the results for the two tests are displayed in Table 1. From the table, two out of 49 results were in full agreement between the two tests. From Additional file 1, all the values measured by the ELISA to be below 1.56 µg/mL (0) were categorized as having levels above 10 µg/mL by the POCT. Which means that the POCT overestimates the SAA levels in serum from lame sows.

**Table 1:** 4 × 4 table for the SAA levels provided by the POCT and ELISA

	ELISA				
		3 (91.8%)^1^	2.5 (0.06%)	2 (0%)	1 (0.02%)
POCT	3	0	0	0	0
	2.5	33	2	0	0
	2	12	1	0	1
	1	0	0	0	0

^1^ The categories corresponded to a serum amyloid A level that is the same for the ELISA and the point-of-care test (Percentage of samples in the corresponding category out of the total number of samples). 3 < 10 µg/mL SAA, 2.5 = 10–50 µg/mL SAA, 2 = 50–400 µg/mL SAA, 1 > 400 µg/mL SAA

## Discussion

In this study, blood from lame untreated gestating sows was used to evaluate the diagnostic performance of two commercially available point-of-care tests. The point-of-care tests were the Cobas b101 CRP test, which is developed for human use, and the OmniChek^TM^-SAA, which is a multispecies lateral flow assay. The correlation of the CRP test was evaluated using Lin’s concordance correlation coefficient, and the agreement was evaluated using a Bland-Altman plot. From the concordance correlation coefficient analysis [20], it is apparent that the POCT is not well correlated to the reference ELISA results and that the ELISA results are higher than the results provided by the POCT. This is also illustrated in the Bland-Altman plot [21], where we have a negative mean difference as well as a trend of increasing differences between the two tests when CRP levels increase. This trend suggested that the underestimation of CRP by the POCT increased with higher CRP levels. The clinical significance of the discrepancy between the two tests can be evaluated based on a threshold of clinical significance of the disease tested, e.g., using a predetermined threshold to classify “positives” and “negatives”. To the authors’ knowledge, no threshold has been established for CRP level and lameness in sows. Using data from studies on CRP levels in healthy and sick pigs [22–24], the threshold between healthy and diseased pigs seems to be between 10 and 70 µg/mL. In this study, the POCT did not measure CRP levels above 40 µg/mL despite having a detection range of 3 to 400 µg/mL, and the ELISA found 14 out of 49 samples above 40 µg/mL. A study evaluating the diagnostic performance of the Cobas b101 CRP-test in humans was not found on the manufacturer’s website, “PubMed”, or “Web of Science”. Hence, it is difficult to determine if the discrepancy is due to poor diagnostic performance of the POCT or lack of compatibility between the CRP antibody used in the assay and porcine CRP. Porcine CRP has been found to partially cross-react with anti-human CRP antibody, as also exploited in another study on immunoassays for CRP [17, 23], and the reference standard used in this study also used anti-human CRP antibodies. Measuring porcine CRP using automated immunoturbidimetric systems developed for human CRP has been validated previously [23, 24]. One of the studies found similar results to the current study [24]. The immunoturbidimetric test showed significant underestimation of CRP levels compared to the reference ELISA and a proportional bias between the two methods. However, in the study, this was corrected by using a porcine serum standard for calibration instead of the human serum standard [24]. Based on the current study, using the Cobas b101 CRP test is currently not advisable in pigs since CRP levels will be underestimated. Using the test as a guide for diagnosing inflammation or antibiotic treatment will result in under-treatment of sick animals. However, if the discrepancy could be solved, the POCT would be a useful tool since the turnaround time is short, the application is easy, and the machine is portable. The OmniChek^TM^-SAA lateral flow assay is marketed as compatible with several species, e.g., pigs, monkeys, rabbits, and seals [25]. The test is semi-quantitative and provides 4 different levels with 1 line corresponding to > 400 µg/mL SAA and 3 lines < 10 µg/mL SAA. The evaluation showed no to poor agreement based on the weighted kappa value of 0.024. The 4 × 4 table showed that the POCT overestimated the level of SAA in the porcine serum samples, with only two samples being correctly classified (category 2.5 = 10–50 µg/mL SAA). A total of 45 out of 49 samples were estimated to be below 10 µg/mL SAA by the ELISA, and only 4 were above. Hence, it is difficult to infer the diagnostic performance across the full range of the POCT in this evaluation study. Having samples with a wider range of SAA levels would have allowed us to determine if the POCT test is able to correctly identify SAA levels above 50 µg/mL. Further, this study intended to evaluate the diagnostic performance of the herd on naturally occurring lameness. The results cannot be extrapolated to other conditions where the SAA levels are higher or more diverse. To fully validate the POCTS, a laboratory validation could be performed; however, that was outside the scope. As with the Cobas b101 CRP test, no studies were found validating the OmniChek^TM^-SAA point-of-care test, and hence, it cannot be determined if the lack of agreement obtained during this study was due to non-compatibility with pig SAA or due to poor performance of the test. Currently, using the POCT to determine SAA levels on sows with lameness would lead to overestimation of SAA levels. If the test was used as an indicator of an inflammatory condition or to guide treatment, overestimation of inflammatory processes and unnecessary treatment could occur. Further validation with a wide range of SAA levels and a laboratory validation would enhance the understanding of the diagnostic performance of the POCTs and their usefulness for the veterinary practitioner.

## Conclusions

Two POCTs, one human CRP test and one multispecies SAA test, were evaluated on porcine blood samples from 49 lame gestating sows. The Cobas b101 CRP test underestimated the CRP levels, and the underestimation seemed to increase with higher CRP levels. No to poor agreement between the OmniChek^TM^-SAA and the reference ELISA was demonstrated, and the SAA POCT overestimated the SAA levels in the blood samples. In conclusion, the two POCTs are currently not suitable for measuring CRP and SAA levels in-herd on blood from lame gestating sows.

## Supplementary Information

Below is the link to the electronic supplementary material.

Supplementary Material 1.

## Data Availability

The datasets used and analysed during the current study are available from the corresponding author on reasonable request.

## Abbreviations

CCC: Lin’s concordance correlation coefficient
CRP: C-reactive protein
Pig-MAP: Pig major acute phase protein
POCT: Point-of-care test
SAA: Serum amyloid A
